# Supplementary figures and images for: Developmental Mapping of Hair Follicles in the Embryonic Stages of Cashmere Goats Using Proteomic and Metabolomic Construction
Source: Animals (Basel). 2023 Sep 30;13(19):3076. doi: 10.3390/ani13193076 (PMC10571814; doi:10.3390/ani13193076)

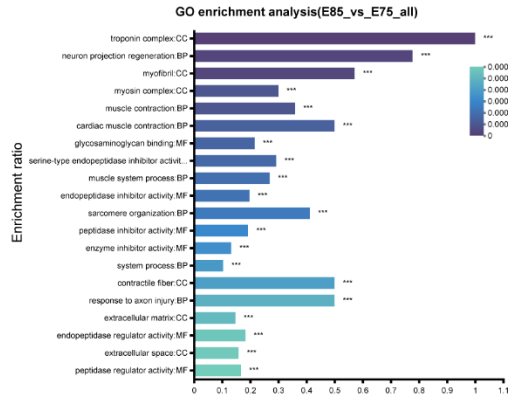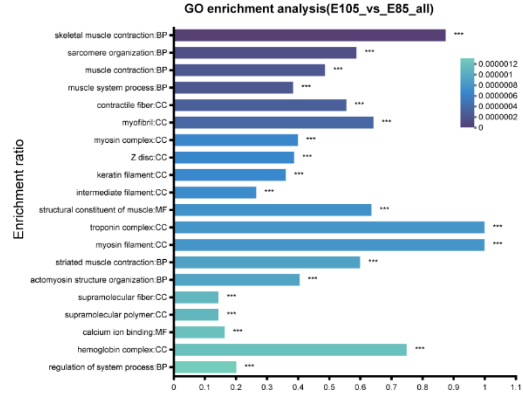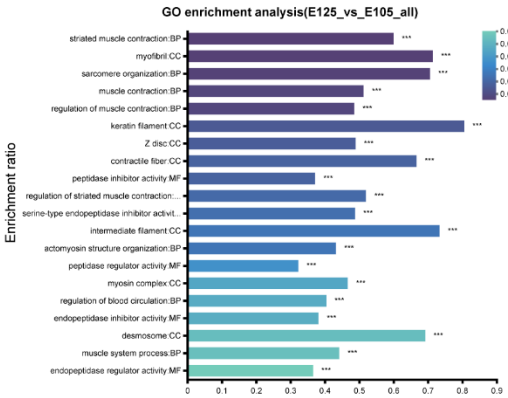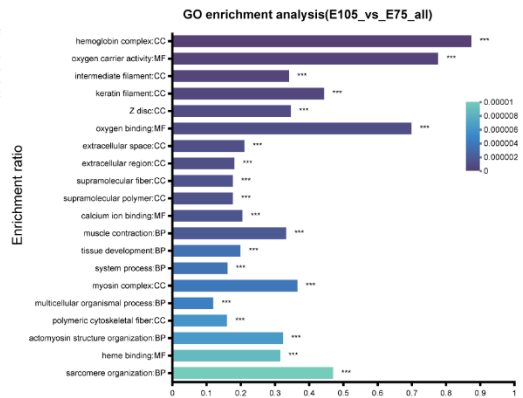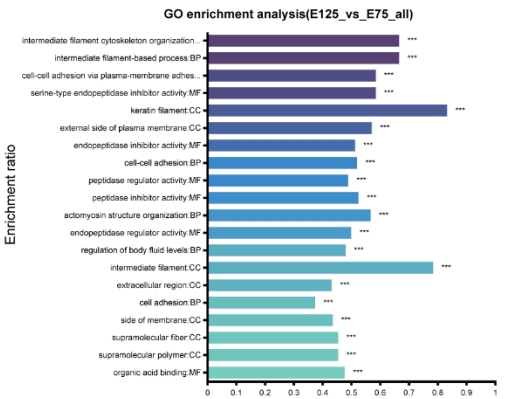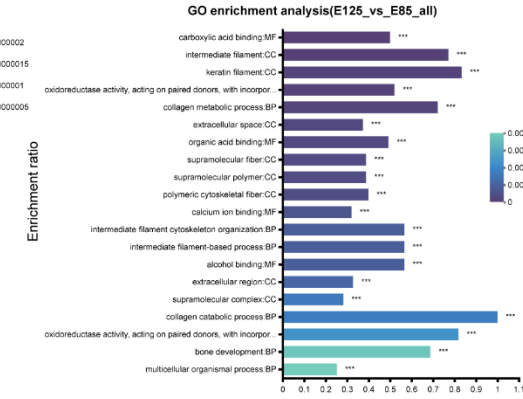

Supplement: Supplementary file 1 [file animals-13-03076-s001.zip › Supplementary Figure S1.pdf]

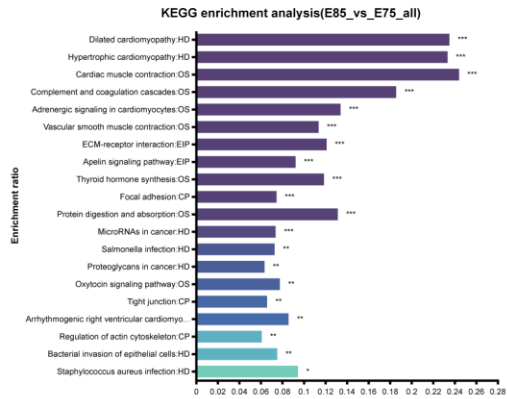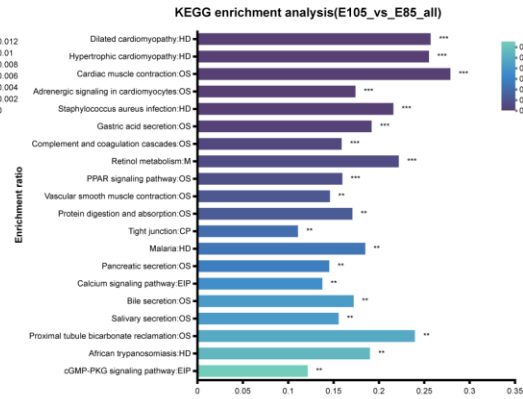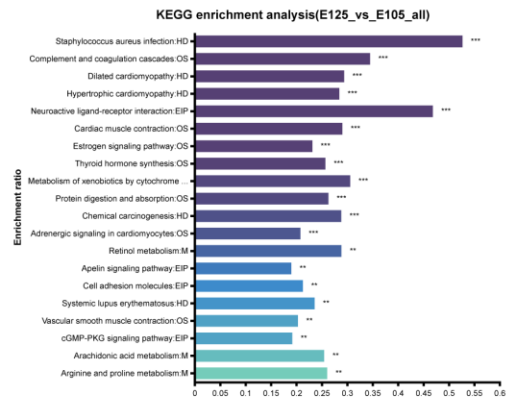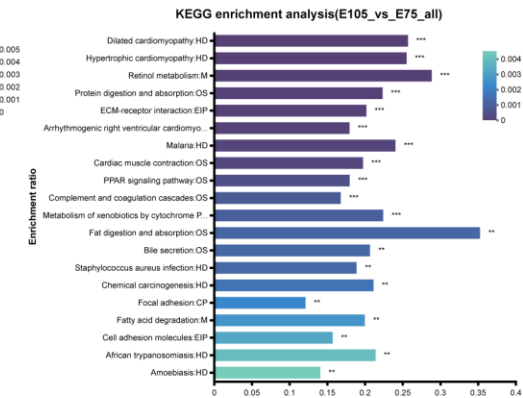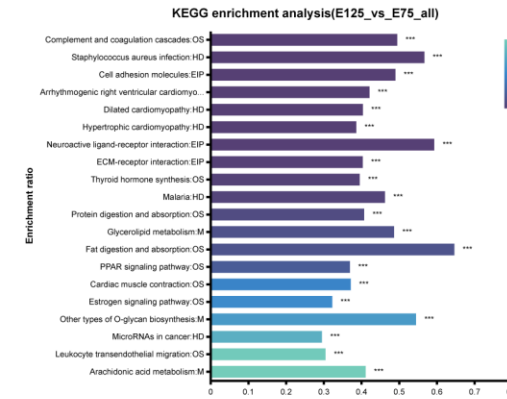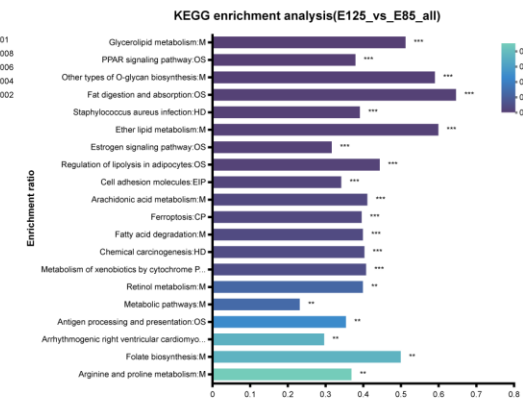

Supplement: Supplementary file 1 [file animals-13-03076-s001.zip › Supplementary Figure S2.pdf]

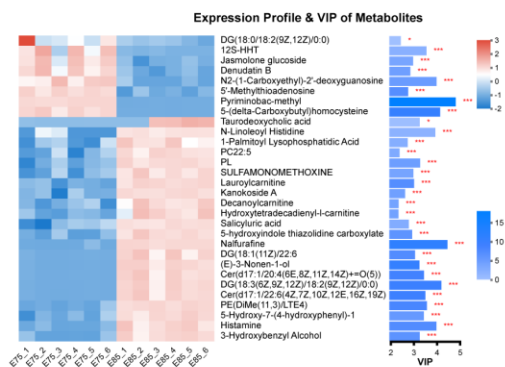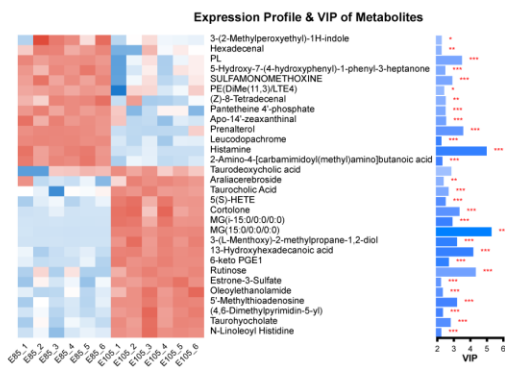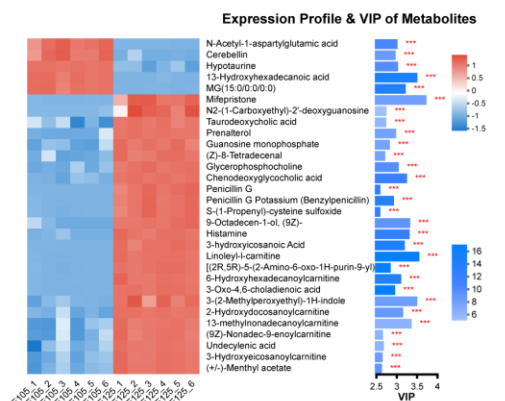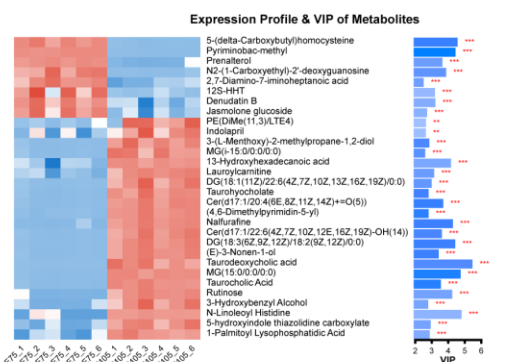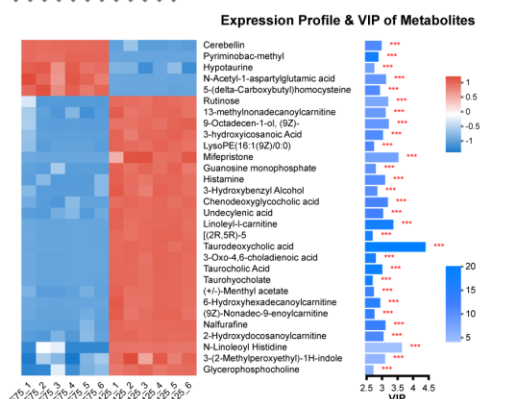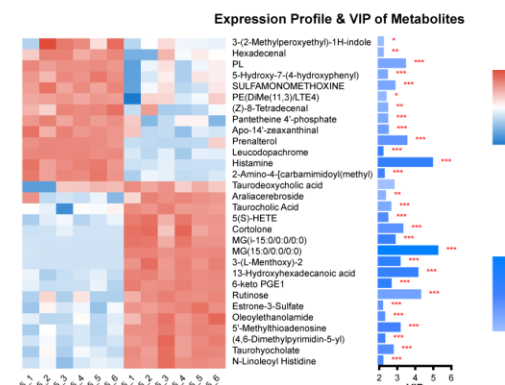

Supplement: Supplementary file 1 [file animals-13-03076-s001.zip › Supplementary Figure S3.pdf]

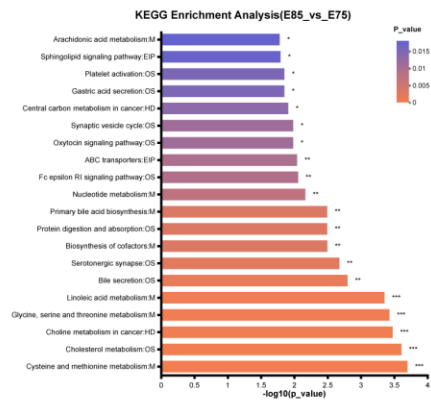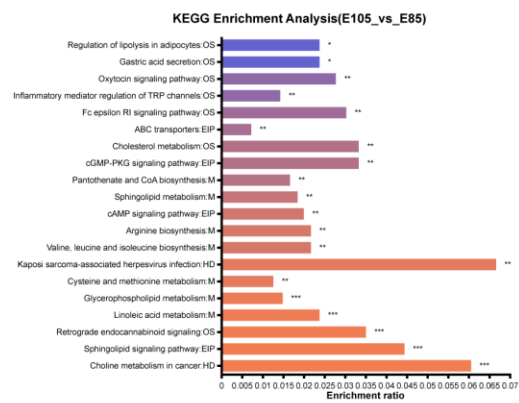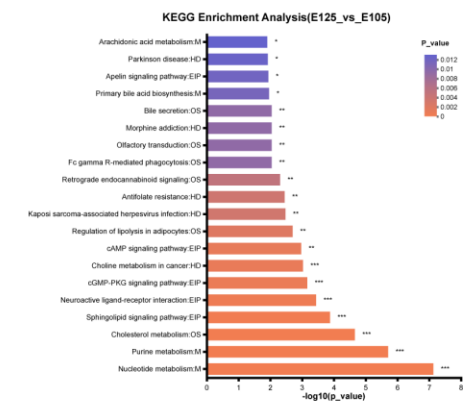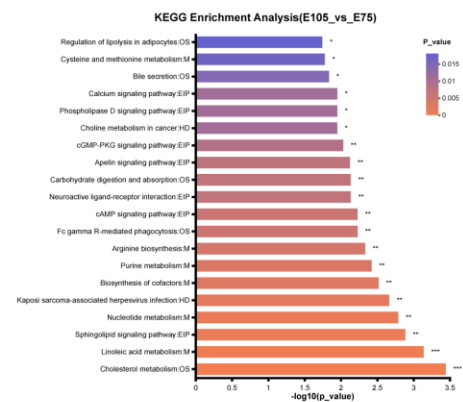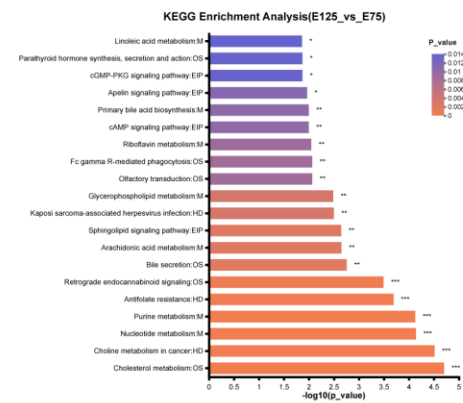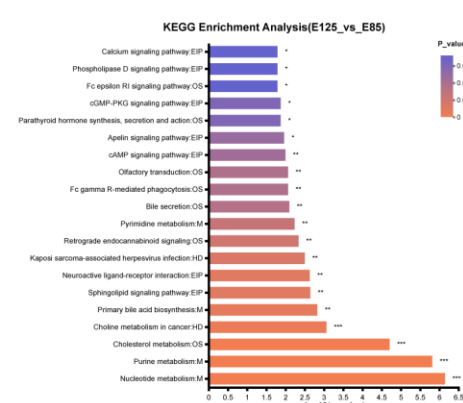

Supplement: Supplementary file 1 [file animals-13-03076-s001.zip › Supplementary Figure S4.pdf]
